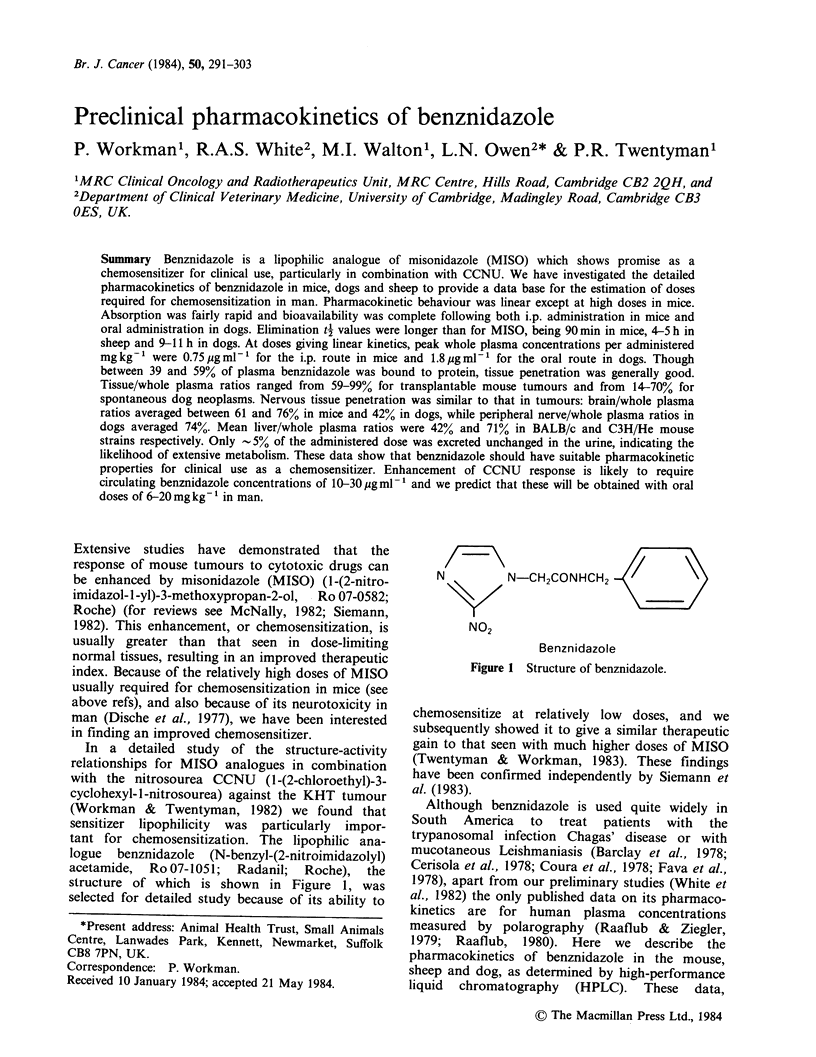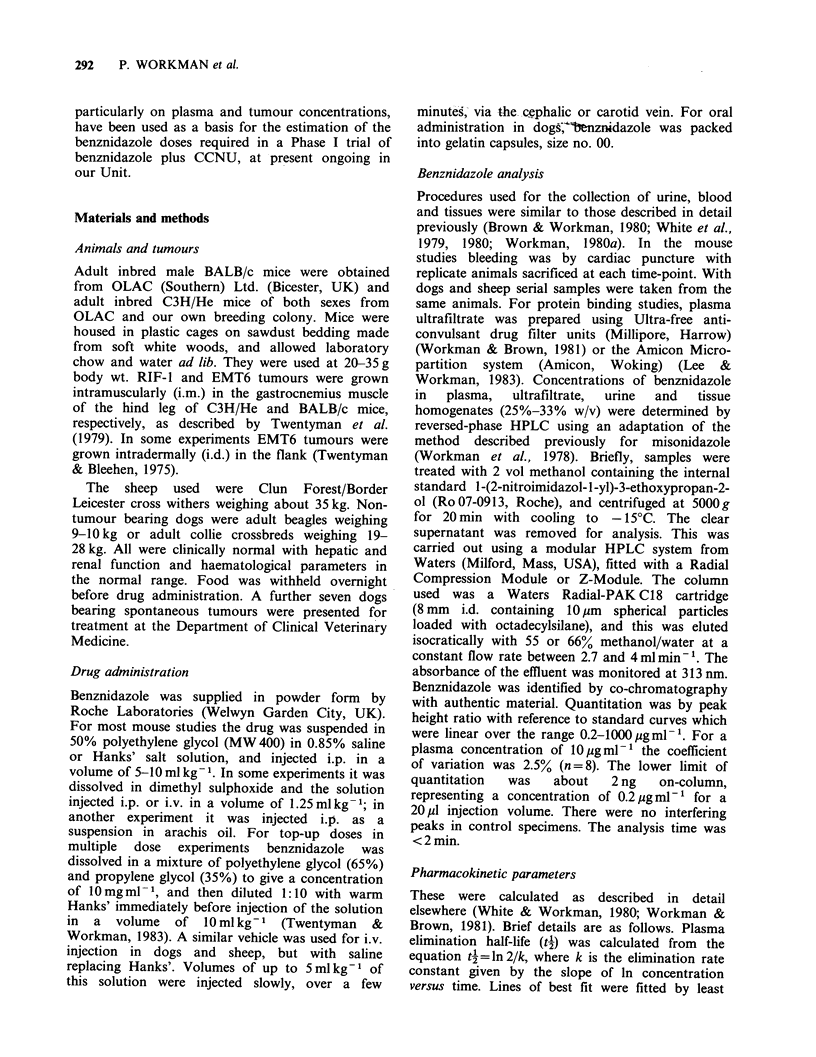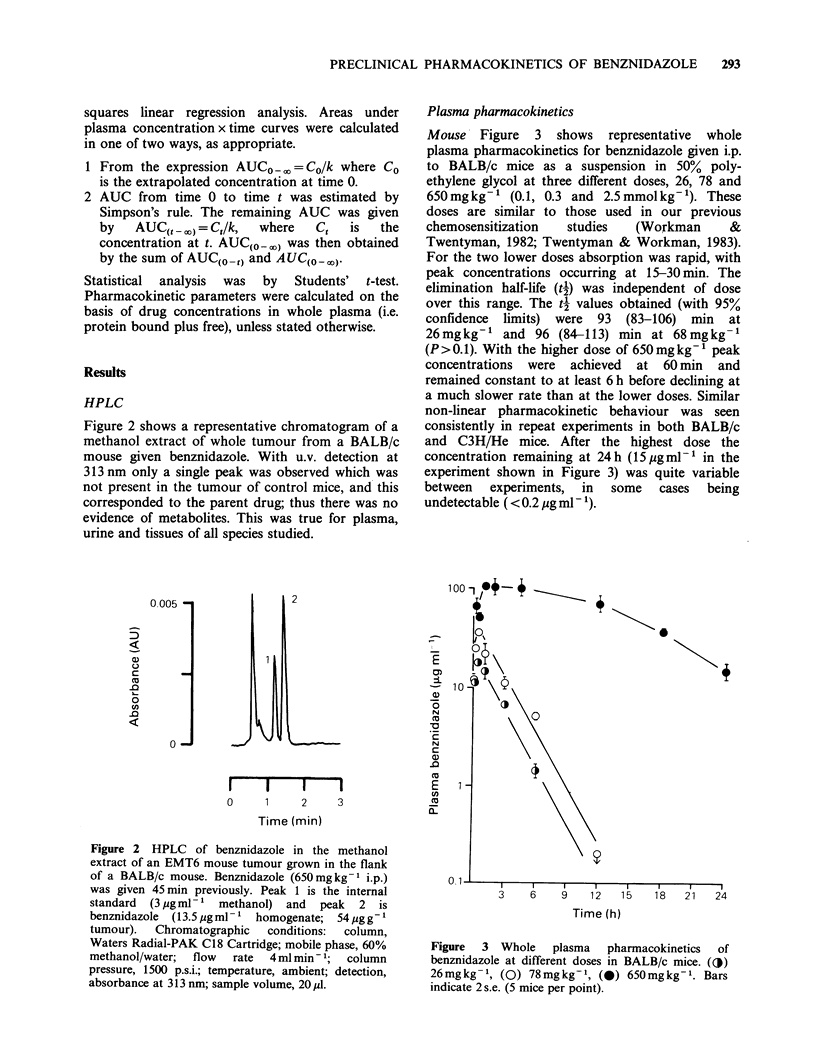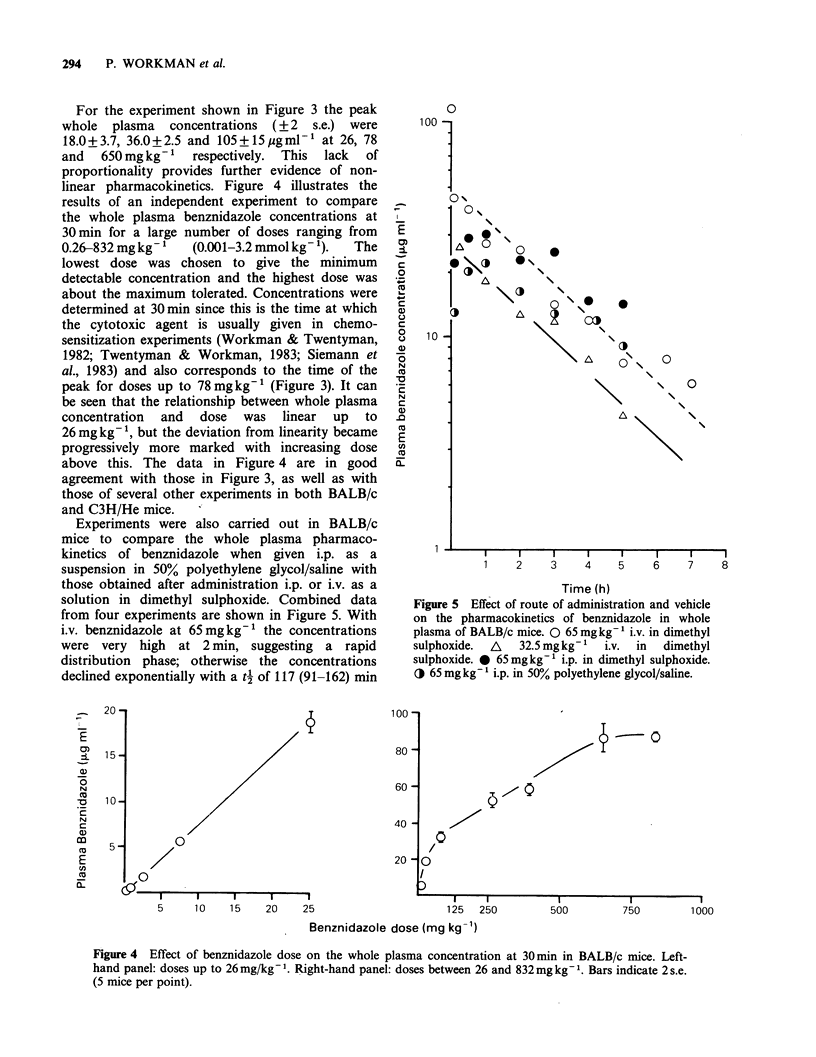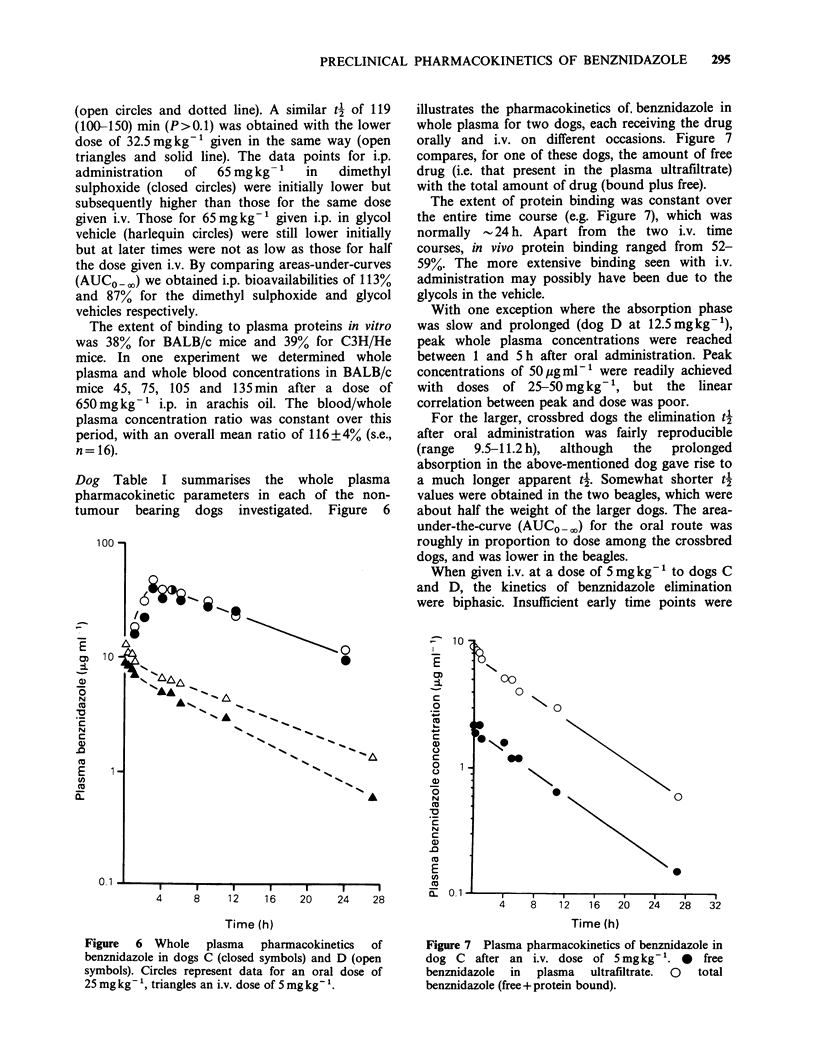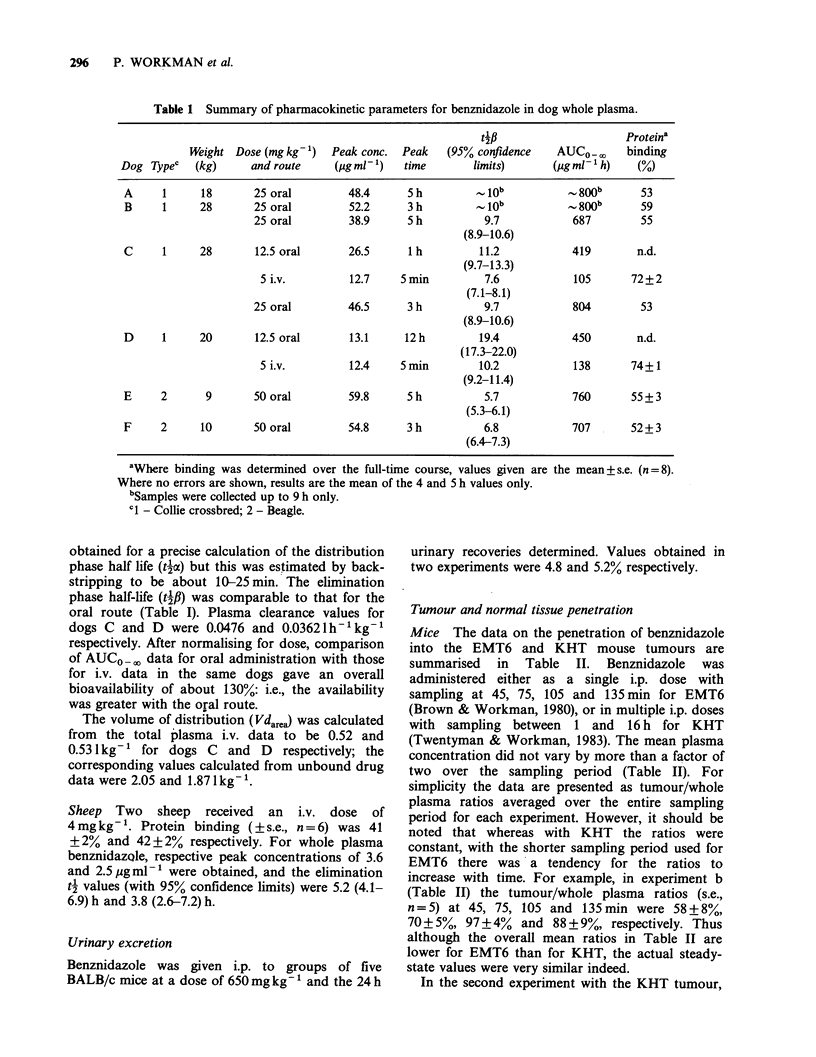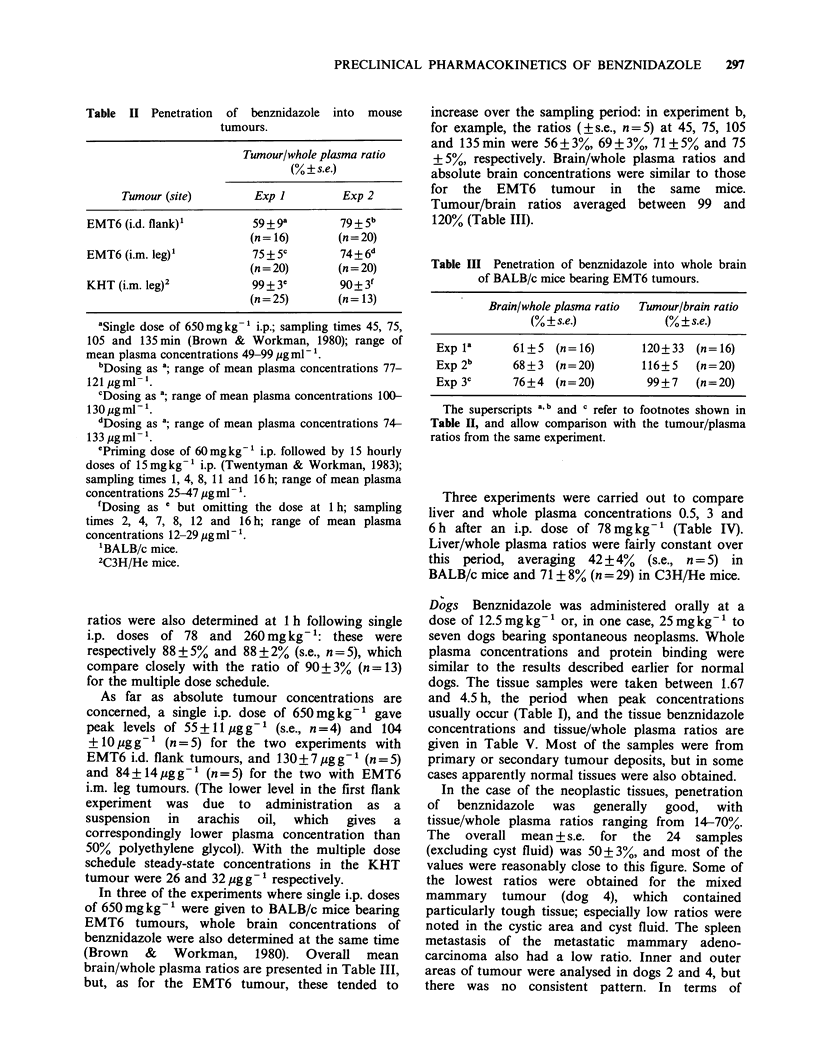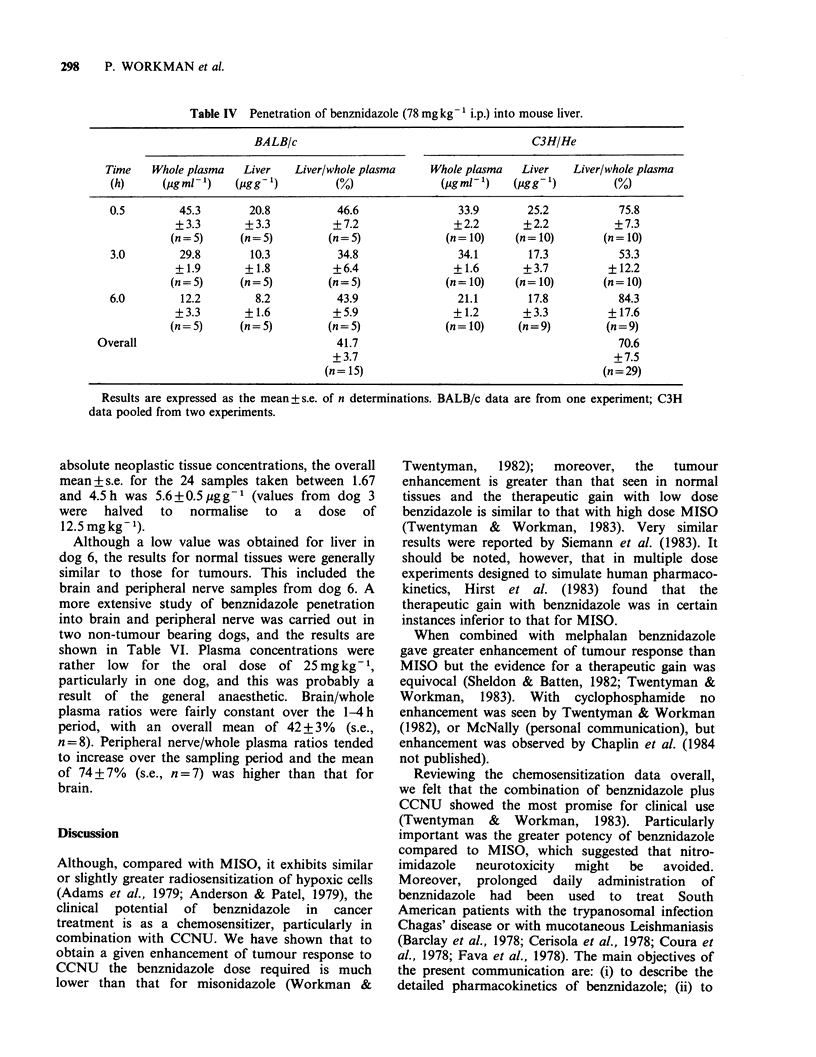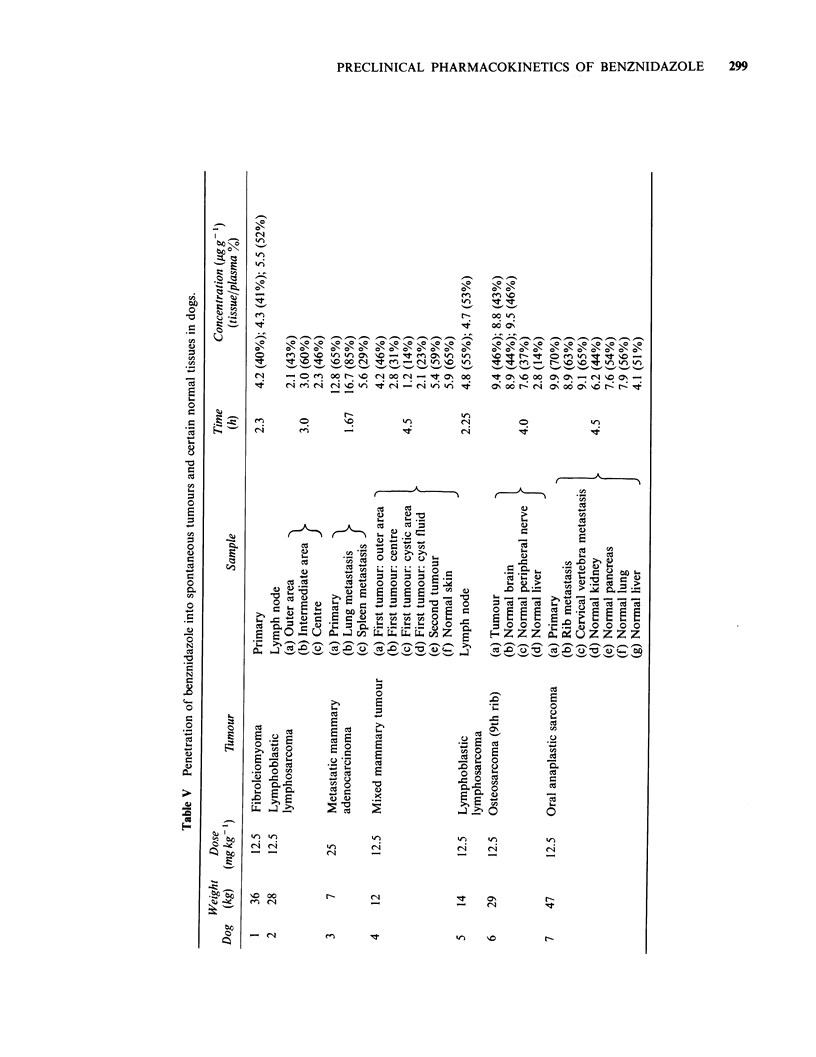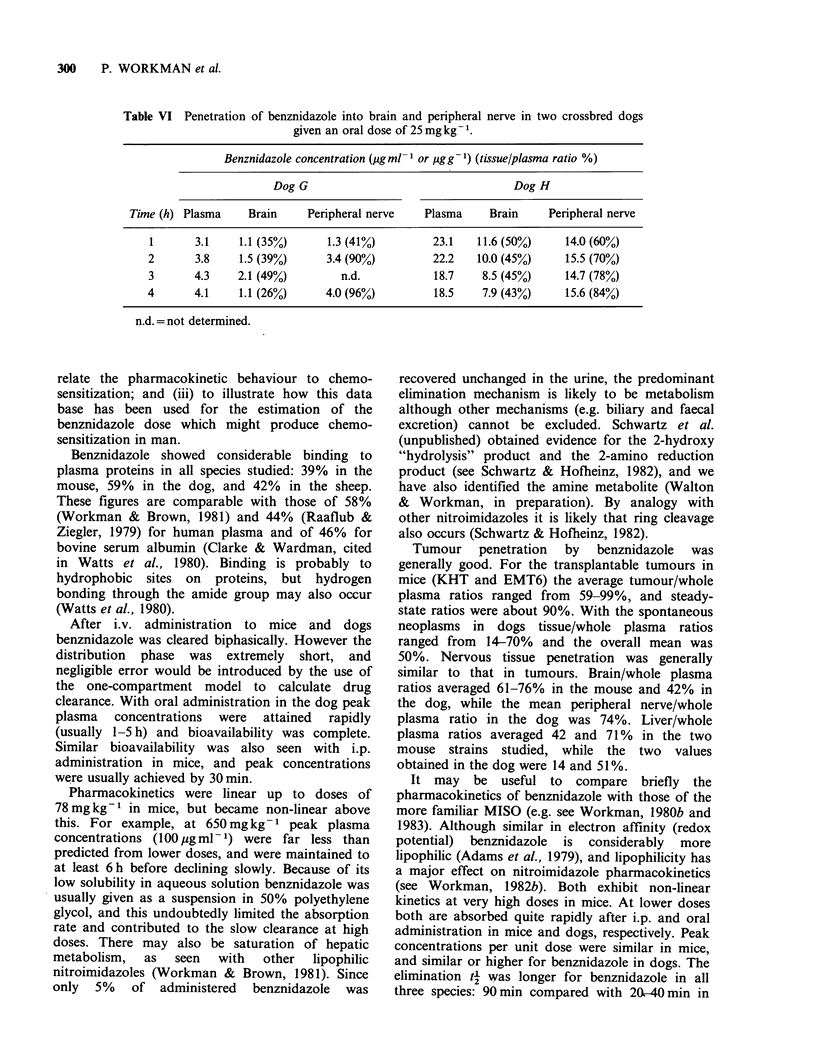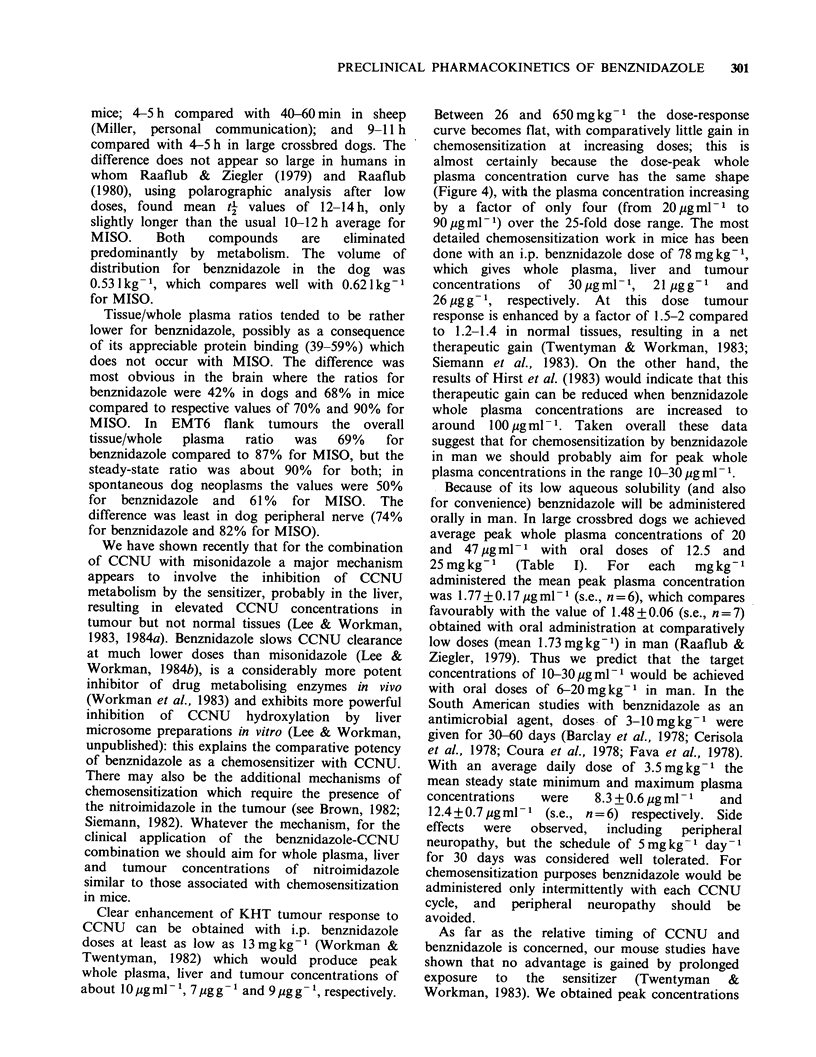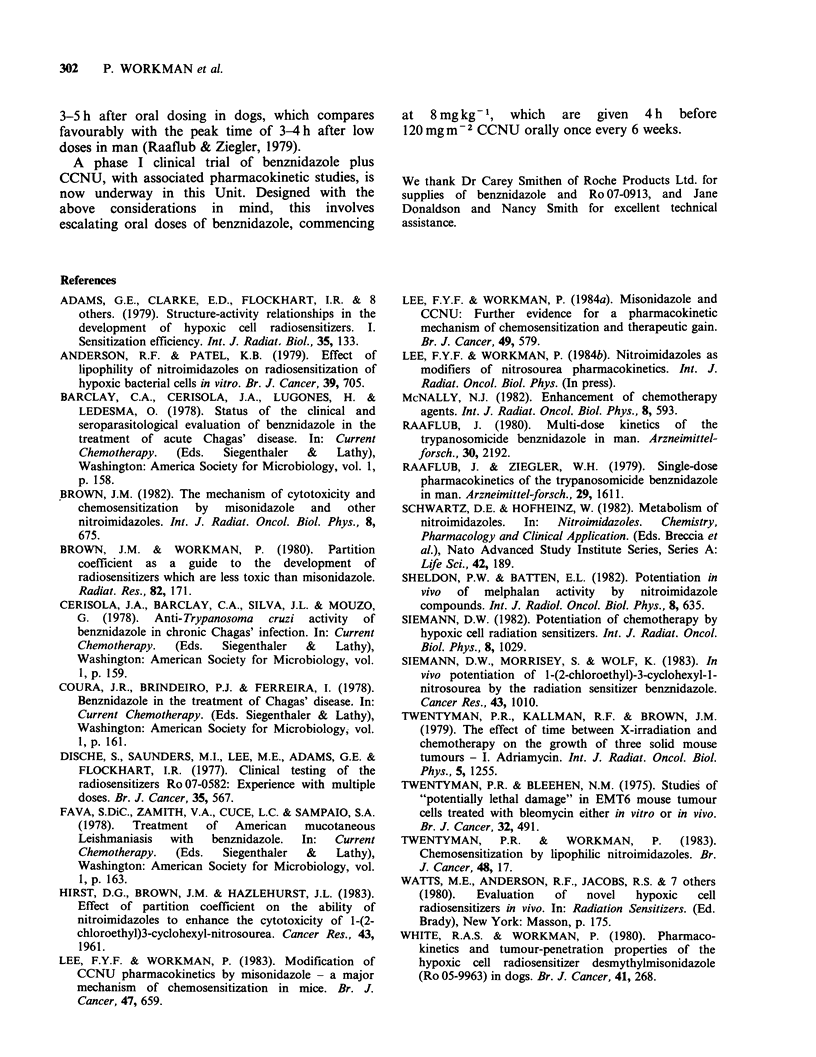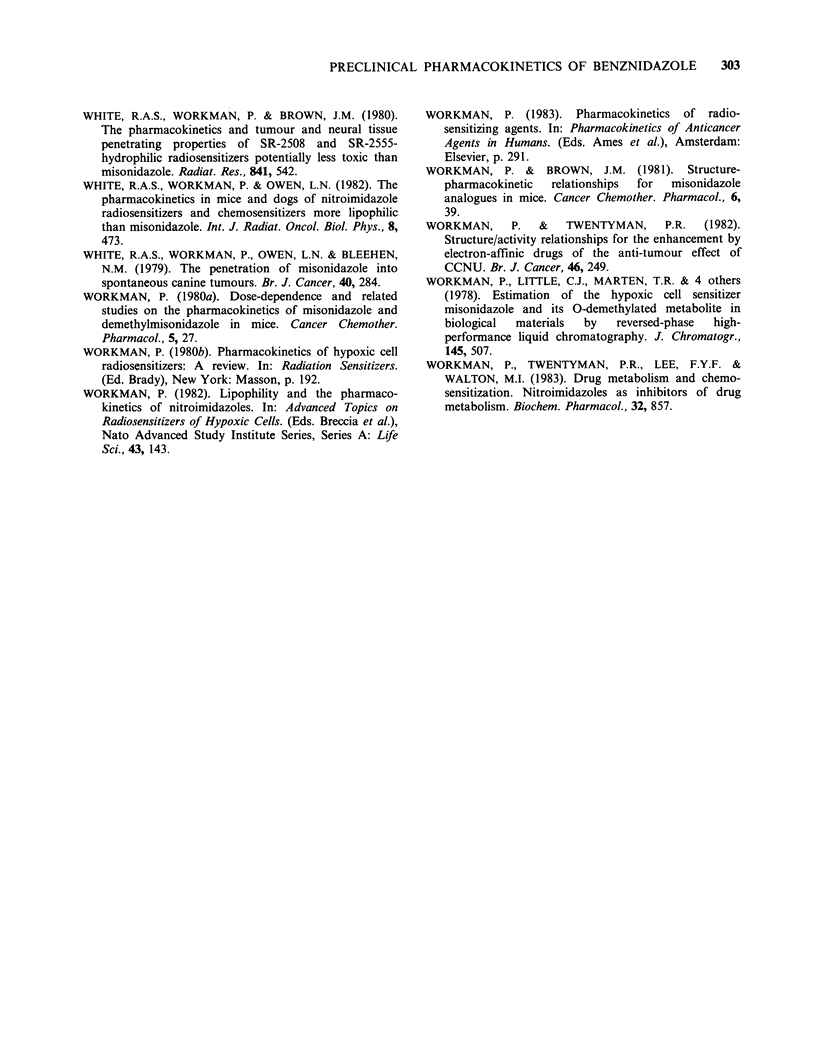# Preclinical pharmacokinetics of benznidazole.

**DOI:** 10.1038/bjc.1984.176

**Published:** 1984-09

**Authors:** P. Workman, R. A. White, M. I. Walton, L. N. Owen, P. R. Twentyman

## Abstract

Benznidazole is a lipophilic analogue of misonidazole (MISO) which shows promise as a chemosensitizer for clinical use, particularly in combination with CCNU. We have investigated the detailed pharmacokinetics of benznidazole in mice, dogs and sheep to provide a data base for the estimation of doses required for chemosensitization in man. Pharmacokinetic behaviour was linear except at high doses in mice. Absorption was fairly rapid and bioavailability was complete following both i.p. administration in mice and oral administration in dogs. Elimination t1/2 values were longer than for MISO, being 90 min in mice, 4-5 h in sheep and 9-11 h in dogs. At doses giving linear kinetics, peak whole plasma concentrations per administered mg kg-1 were 0.75 micrograms ml-1 for the i.p. route in mice and 1.8 micrograms ml-1 for the oral route in dogs. Though between 39 and 59% of plasma benznidazole was bound to protein, tissue penetration was generally good. Tissue/whole plasma ratios ranged from 59-99% for transplantable mouse tumours and from 14-70% for spontaneous dog neoplasms. Nervous tissue penetration was similar to that in tumours: brain/whole plasma ratios averaged between 61 and 76% in mice and 42% in dogs, while peripheral nerve/whole plasma ratios in dogs averaged 74%. Mean liver/whole plasma ratios were 42% and 71% in BALB/c and C3H/He mouse strains respectively. Only approximately 5% of the administered dose was excreted unchanged in the urine, indicating the likelihood of extensive metabolism. These data show that benznidazole should have suitable pharmacokinetic properties for clinical use as a chemosensitizer. Enhancement of CCNU response is likely to require circulating benznidazole concentrations of 10-30 micrograms ml-1 and we predict that these will be obtained with oral doses of 6-20 mg kg-1 in man.


Br. J. Cancer (1984), **50**, 291–303

### Preclinical pharmacokinetics of benznidazole

P. Workman[1], R.A.S. White[2], M.I. Walton[1], L.N. Owen[2]* & P.R. Twentyman[1]

[1]*MRC Clinical Oncology and Radiotherapeutics Unit, MRC Centre, Hills Road, Cambridge CB2 2QH, and* [2]*Department of Clinical Veterinary Medicine, University of Cambridge, Madingley Road, Cambridge CB3 0ES, UK.*

**Summary** Benznidazole is a lipophilic analogue of misonidazole (MISO) which shows promise as a chemosensitizer for clinical use, particularly in combination with CCNU. We have investigated the detailed pharmacokinetics of benznidazole in mice, dogs and sheep to provide a data base for the estimation of doses required for chemosensitization in man. Pharmacokinetic behaviour was linear except at high doses in mice. Absorption was fairly rapid and bioavailability was complete following both i.p. administration in mice and oral administration in dogs. Elimination $t_{\frac{1}{2}}$ values were longer than for MISO, being 90 min in mice, 4–5 h in sheep and 9–11 h in dogs. At doses giving linear kinetics, peak whole plasma concentrations per administered $mg\,kg^{-1}$ were $0.75\,\mu g\,ml^{-1}$ for the i.p. route in mice and $1.8\,\mu g\,ml^{-1}$ for the oral route in dogs. Though between 39 and 59% of plasma benznidazole was bound to protein, tissue penetration was generally good. Tissue/whole plasma ratios ranged from 59–99% for transplantable mouse tumours and from 14–70% for spontaneous dog neoplasms. Nervous tissue penetration was similar to that in tumours: brain/whole plasma ratios averaged between 61 and 76% in mice and 42% in dogs, while peripheral nerve/whole plasma ratios in dogs averaged 74%. Mean liver/whole plasma ratios were 42% and 71% in BALB/c and C3H/He mouse strains respectively. Only ~5% of the administered dose was excreted unchanged in the urine, indicating the likelihood of extensive metabolism. These data show that benznidazole should have suitable pharmacokinetic properties for clinical use as a chemosensitizer. Enhancement of CCNU response is likely to require circulating benznidazole concentrations of $10–30\,\mu g\,ml^{-1}$ and we predict that these will be obtained with oral doses of $6–20\,mg\,kg^{-1}$ in man.

Extensive studies have demonstrated that the response of mouse tumours to cytotoxic drugs can be enhanced by misonidazole (MISO) (1-(2-nitro-imidazol-1-yl)-3-methoxypropan-2-ol, Ro 07-0582; Roche) (for reviews see McNally, 1982; Siemann, 1982). This enhancement, or chemosensitization, is usually greater than that seen in dose-limiting normal tissues, resulting in an improved therapeutic index. Because of the relatively high doses of MISO usually required for chemosensitization in mice (see above refs), and also because of its neurotoxicity in man (Dische *et al.*, 1977), we have been interested in finding an improved chemosensitizer.

In a detailed study of the structure-activity relationships for MISO analogues in combination with the nitrosourea CCNU (1-(2-chloroethyl)-3-cyclohexyl-1-nitrosourea) against the KHT tumour (Workman & Twentyman, 1982) we found that sensitizer lipophilicity was particularly important for chemosensitization. The lipophilic analogue benznidazole (N-benzyl-(2-nitroimidazolyl) acetamide, Ro 07-1051; Radanil; Roche), the structure of which is shown in Figure 1, was selected for detailed study because of its ability to

**Figure 1** Structure of benznidazole.

chemosensitize at relatively low doses, and we subsequently showed it to give a similar therapeutic gain to that seen with much higher doses of MISO (Twentyman & Workman, 1983). These findings have been confirmed independently by Siemann *et al.* (1983).

Although benznidazole is used quite widely in South America to treat patients with the trypanosomal infection Chagas' disease or with mucotaneous Leishmaniasis (Barclay *et al.*, 1978; Cerisola *et al.*, 1978; Coura *et al.*, 1978; Fava *et al.*, 1978), apart from our preliminary studies (White *et al.*, 1982) the only published data on its pharmacokinetics are for human plasma concentrations measured by polarography (Raaflub & Ziegler, 1979; Raaflub, 1980). Here we describe the pharmacokinetics of benznidazole in the mouse, sheep and dog, as determined by high-performance liquid chromatography (HPLC). These data,

*Present address: Animal Health Trust, Small Animals Centre, Lanwades Park, Kennett, Newmarket, Suffolk CB8 7PN, UK.
Correspondence: P. Workman.
Received 10 January 1984; accepted 21 May 1984.

© The Macmillan Press Ltd., 1984

particularly on plasma and tumour concentrations, have been used as a basis for the estimation of the benznidazole doses required in a Phase I trial of benznidazole plus CCNU, at present ongoing in our Unit.

### Materials and methods

### *Animals and tumours*

Adult inbred male BALB/c mice were obtained from OLAC (Southern) Ltd. (Bicester, UK) and adult inbred C3H/He mice of both sexes from OLAC and our own breeding colony. Mice were housed in plastic cages on sawdust bedding made from soft white woods, and allowed laboratory chow and water *ad lib*. They were used at 20–35 g body wt. RIF-1 and EMT6 tumours were grown intramuscularly (i.m.) in the gastrocnemius muscle of the hind leg of C3H/He and BALB/c mice, respectively, as described by Twentyman *et al.* (1979). In some experiments EMT6 tumours were grown intradermally (i.d.) in the flank (Twentyman & Bleehen, 1975).

The sheep used were Clun Forest/Border Leicester cross withers weighing about 35 kg. Non-tumour bearing dogs were adult beagles weighing 9–10 kg or adult collie crossbreds weighing 19–28 kg. All were clinically normal with hepatic and renal function and haematological parameters in the normal range. Food was withheld overnight before drug administration. A further seven dogs bearing spontaneous tumours were presented for treatment at the Department of Clinical Veterinary Medicine.

### *Drug administration*

Benznidazole was supplied in powder form by Roche Laboratories (Welwyn Garden City, UK). For most mouse studies the drug was suspended in 50% polyethylene glycol (MW 400) in 0.85% saline or Hanks' salt solution, and injected i.p. in a volume of 5–10 ml kg$^{-1}$. In some experiments it was dissolved in dimethyl sulphoxide and the solution injected i.p. or i.v. in a volume of 1.25 ml kg$^{-1}$; in another experiment it was injected i.p. as a suspension in arachis oil. For top-up doses in multiple dose experiments benznidazole was dissolved in a mixture of polyethylene glycol (65%) and propylene glycol (35%) to give a concentration of 10 mg ml$^{-1}$, and then diluted 1:10 with warm Hanks' immediately before injection of the solution in a volume of 10 ml kg$^{-1}$ (Twentyman & Workman, 1983). A similar vehicle was used for i.v. injection in dogs and sheep, but with saline replacing Hanks'. Volumes of up to 5 ml kg$^{-1}$ of this solution were injected slowly, over a few minutes, via the cephalic or carotid vein. For oral administration in dogs, benznidazole was packed into gelatin capsules, size no. 00.

### *Benznidazole analysis*

Procedures used for the collection of urine, blood and tissues were similar to those described in detail previously (Brown & Workman, 1980; White *et al.*, 1979, 1980; Workman, 1980*a*). In the mouse studies bleeding was by cardiac puncture with replicate animals sacrificed at each time-point. With dogs and sheep serial samples were taken from the same animals. For protein binding studies, plasma ultrafiltrate was prepared using Ultra-free anti-convulsant drug filter units (Millipore, Harrow) (Workman & Brown, 1981) or the Amicon Micro-partition system (Amicon, Woking) (Lee & Workman, 1983). Concentrations of benznidazole in plasma, ultrafiltrate, urine and tissue homogenates (25%–33% w/v) were determined by reversed-phase HPLC using an adaptation of the method described previously for misonidazole (Workman *et al.*, 1978). Briefly, samples were treated with 2 vol methanol containing the internal standard 1-(2-nitroimidazol-1-yl)-3-ethoxypropan-2-ol (Ro 07-0913, Roche), and centrifuged at 5000 *g* for 20 min with cooling to $-15$°C. The clear supernatant was removed for analysis. This was carried out using a modular HPLC system from Waters (Milford, Mass, USA), fitted with a Radial Compression Module or Z-Module. The column used was a Waters Radial-PAK C18 cartridge (8 mm i.d. containing 10 $\mu$m spherical particles loaded with octadecylsilane), and this was eluted isocratically with 55 or 66% methanol/water at a constant flow rate between 2.7 and 4 ml min$^{-1}$. The absorbance of the effluent was monitored at 313 nm. Benznidazole was identified by co-chromatography with authentic material. Quantitation was by peak height ratio with reference to standard curves which were linear over the range 0.2–1000 $\mu$g ml$^{-1}$. For a plasma concentration of 10 $\mu$g ml$^{-1}$ the coefficient of variation was 2.5% ($n=8$). The lower limit of quantitation was about 2 ng on-column, representing a concentration of 0.2 $\mu$g ml$^{-1}$ for a 20 $\mu$l injection volume. There were no interfering peaks in control specimens. The analysis time was $<2$ min.

### *Pharmacokinetic parameters*

These were calculated as described in detail elsewhere (White & Workman, 1980; Workman & Brown, 1981). Brief details are as follows. Plasma elimination half-life ($t\frac{1}{2}$) was calculated from the equation $t\frac{1}{2} = \ln 2/k$, where $k$ is the elimination rate constant given by the slope of ln concentration *versus* time. Lines of best fit were fitted by least squares linear regression analysis. Areas under plasma concentration × time curves were calculated in one of two ways, as appropriate.

1 From the expression $AUC_{0-\infty}=C_0/k$ where $C_0$ is the extrapolated concentration at time 0.
2 AUC from time 0 to time $t$ was estimated by Simpson's rule. The remaining AUC was given by $AUC_{(t-\infty)}=C_t/k$, where $C_t$ is the concentration at $t$. $AUC_{(0-\infty)}$ was then obtained by the sum of $AUC_{(0-t)}$ and $AUC_{(0-\infty)}$.

Statistical analysis was by Students' $t$-test. Pharmacokinetic parameters were calculated on the basis of drug concentrations in whole plasma (i.e. protein bound plus free), unless stated otherwise.

### Results

### *HPLC*

Figure 2 shows a representative chromatogram of a methanol extract of whole tumour from a BALB/c mouse given benznidazole. With u.v. detection at 313 nm only a single peak was observed which was not present in the tumour of control mice, and this corresponded to the parent drug; thus there was no evidence of metabolites. This was true for plasma, urine and tissues of all species studied.

### *Plasma pharmacokinetics*

*Mouse* Figure 3 shows representative whole plasma pharmacokinetics for benznidazole given i.p. to BALB/c mice as a suspension in 50% polyethylene glycol at three different doses, 26, 78 and 650 mg kg$^{-1}$ (0.1, 0.3 and 2.5 mmol kg$^{-1}$). These doses are similar to those used in our previous chemosensitization studies (Workman & Twentyman, 1982; Twentyman & Workman, 1983). For the two lower doses absorption was rapid, with peak concentrations occurring at 15–30 min. The elimination half-life ($t\frac{1}{2}$) was independent of dose over this range. The $t\frac{1}{2}$ values obtained (with 95% confidence limits) were 93 (83–106) min at 26 mg kg$^{-1}$ and 96 (84–113) min at 68 mg kg$^{-1}$ ($P>0.1$). With the higher dose of 650 mg kg$^{-1}$ peak concentrations were achieved at 60 min and remained constant to at least 6 h before declining at a much slower rate than at the lower doses. Similar non-linear pharmacokinetic behaviour was seen consistently in repeat experiments in both BALB/c and C3H/He mice. After the highest dose the concentration remaining at 24 h (15 $\mu$g ml$^{-1}$ in the experiment shown in Figure 3) was quite variable between experiments, in some cases being undetectable (<0.2 $\mu$g ml$^{-1}$).

**Figure 2** HPLC of benznidazole in the methanol extract of an EMT6 mouse tumour grown in the flank of a BALB/c mouse. Benznidazole (650 mg kg$^{-1}$ i.p.) was given 45 min previously. Peak 1 is the internal standard (3 $\mu$g ml$^{-1}$ methanol) and peak 2 is benznidazole (13.5 $\mu$g ml$^{-1}$ homogenate; 54 $\mu$g g$^{-1}$ tumour). Chromatographic conditions: column, Waters Radial-PAK C18 Cartridge; mobile phase, 60% methanol/water; flow rate 4 ml min$^{-1}$; column pressure, 1500 p.s.i.; temperature, ambient; detection, absorbance at 313 nm; sample volume, 20 $\mu$l.

**Figure 3** Whole plasma pharmacokinetics of benznidazole at different doses in BALB/c mice. (◑) 26 mg kg$^{-1}$, (○) 78 mg kg$^{-1}$, (●) 650 mg kg$^{-1}$. Bars indicate 2 s.e. (5 mice per point).

For the experiment shown in Figure 3 the peak whole plasma concentrations ($\pm 2$ s.e.) were $18.0 \pm 3.7$, $36.0 \pm 2.5$ and $105 \pm 15\,\mu g\,ml^{-1}$ at 26, 78 and $650\,mg\,kg^{-1}$ respectively. This lack of proportionality provides further evidence of non-linear pharmacokinetics. Figure 4 illustrates the results of an independent experiment to compare the whole plasma benznidazole concentrations at 30 min for a large number of doses ranging from $0.26$–$832\,mg\,kg^{-1}$ ($0.001$–$3.2\,mmol\,kg^{-1}$). The lowest dose was chosen to give the minimum detectable concentration and the highest dose was about the maximum tolerated. Concentrations were determined at 30 min since this is the time at which the cytotoxic agent is usually given in chemosensitization experiments (Workman & Twentyman, 1982; Twentyman & Workman, 1983; Siemann *et al.*, 1983) and also corresponds to the time of the peak for doses up to $78\,mg\,kg^{-1}$ (Figure 3). It can be seen that the relationship between whole plasma concentration and dose was linear up to $26\,mg\,kg^{-1}$, but the deviation from linearity became progressively more marked with increasing dose above this. The data in Figure 4 are in good agreement with those in Figure 3, as well as with those of several other experiments in both BALB/c and C3H/He mice.

Experiments were also carried out in BALB/c mice to compare the whole plasma pharmacokinetics of benznidazole when given i.p. as a suspension in 50% polyethylene glycol/saline with those obtained after administration i.p. or i.v. as a solution in dimethyl sulphoxide. Combined data from four experiments are shown in Figure 5. With i.v. benznidazole at $65\,mg\,kg^{-1}$ the concentrations were very high at 2 min, suggesting a rapid distribution phase; otherwise the concentrations declined exponentially with a $t_{\frac{1}{2}}$ of 117 (91–162) min

**Figure 5** Effect of route of administration and vehicle on the pharmacokinetics of benznidazole in whole plasma of BALB/c mice. ○ $65\,mg\,kg^{-1}$ i.v. in dimethyl sulphoxide. △ $32.5\,mg\,kg^{-1}$ i.v. in dimethyl sulphoxide. ● $65\,mg\,kg^{-1}$ i.p. in dimethyl sulphoxide. ◑ $65\,mg\,kg^{-1}$ i.p. in 50% polyethylene glycol/saline.

**Figure 4** Effect of benznidazole dose on the whole plasma concentration at 30 min in BALB/c mice. Left-hand panel: doses up to $26\,mg/kg^{-1}$. Right-hand panel: doses between 26 and $832\,mg\,kg^{-1}$. Bars indicate 2 s.e. (5 mice per point).

(open circles and dotted line). A similar $t\frac{1}{2}$ of 119 (100–150) min ($P>0.1$) was obtained with the lower dose of 32.5 mg kg$^{-1}$ given in the same way (open triangles and solid line). The data points for i.p. administration of 65 mg kg$^{-1}$ in dimethyl sulphoxide (closed circles) were initially lower but subsequently higher than those for the same dose given i.v. Those for 65 mg kg$^{-1}$ given i.p. in glycol vehicle (harlequin circles) were still lower initially but at later times were not as low as those for half the dose given i.v. By comparing areas-under-curves ($AUC_{0-\infty}$) we obtained i.p. bioavailabilities of 113% and 87% for the dimethyl sulphoxide and glycol vehicles respectively.

The extent of binding to plasma proteins *in vitro* was 38% for BALB/c mice and 39% for C3H/He mice. In one experiment we determined whole plasma and whole blood concentrations in BALB/c mice 45, 75, 105 and 135 min after a dose of 650 mg kg$^{-1}$ i.p. in arachis oil. The blood/whole plasma concentration ratio was constant over this period, with an overall mean ratio of 116±4% (s.e., $n=16$).

*Dog* Table I summarises the whole plasma pharmacokinetic parameters in each of the non-tumour bearing dogs investigated. Figure 6 illustrates the pharmacokinetics of. benznidazole in whole plasma for two dogs, each receiving the drug orally and i.v. on different occasions. Figure 7 compares, for one of these dogs, the amount of free drug (i.e. that present in the plasma ultrafiltrate) with the total amount of drug (bound plus free).

The extent of protein binding was constant over the entire time course (e.g. Figure 7), which was normally ~24 h. Apart from the two i.v. time courses, *in vivo* protein binding ranged from 52–59%. The more extensive binding seen with i.v. administration may possibly have been due to the glycols in the vehicle.

With one exception where the absorption phase was slow and prolonged (dog D at 12.5 mg kg$^{-1}$), peak whole plasma concentrations were reached between 1 and 5 h after oral administration. Peak concentrations of 50 μg ml$^{-1}$ were readily achieved with doses of 25–50 mg kg$^{-1}$, but the linear correlation between peak and dose was poor.

For the larger, crossbred dogs the elimination $t\frac{1}{2}$ after oral administration was fairly reproducible (range 9.5–11.2 h), although the prolonged absorption in the above-mentioned dog gave rise to a much longer apparent $t\frac{1}{2}$. Somewhat shorter $t\frac{1}{2}$ values were obtained in the two beagles, which were about half the weight of the larger dogs. The area-under-the-curve ($AUC_{0-\infty}$) for the oral route was roughly in proportion to dose among the crossbred dogs, and was lower in the beagles.

When given i.v. at a dose of 5 mg kg$^{-1}$ to dogs C and D, the kinetics of benznidazole elimination were biphasic. Insufficient early time points were

**Figure 6** Whole plasma pharmacokinetics of benznidazole in dogs C (closed symbols) and D (open symbols). Circles represent data for an oral dose of 25 mg kg$^{-1}$, triangles an i.v. dose of 5 mg kg$^{-1}$.

**Figure 7** Plasma pharmacokinetics of benznidazole in dog C after an i.v. dose of 5 mg kg$^{-1}$. ● free benznidazole in plasma ultrafiltrate. ○ total benznidazole (free + protein bound).

**Table 1** Summary of pharmacokinetic parameters for benznidazole in dog whole plasma.

| Dog | Type[c] | Weight (kg) | Dose ($mg\,kg^{-1}$) and route | Peak conc. ($\mu g\,ml^{-1}$) | Peak time | $t\frac{1}{2}\beta$ (95% confidence limits) | $AUC_{0-\infty}$ ($\mu g\,ml^{-1}\,h$) | Protein[a] binding (%) |
|---|---|---|---|---|---|---|---|---|
| A | 1 | 18 | 25 oral | 48.4 | 5 h | ~10[b] | ~800[b] | 53 |
| B | 1 | 28 | 25 oral | 52.2 | 3 h | ~10[b] | ~800[b] | 59 |
| | | | 25 oral | 38.9 | 5 h | 9.7 (8.9–10.6) | 687 | 55 |
| C | 1 | 28 | 12.5 oral | 26.5 | 1 h | 11.2 (9.7–13.3) | 419 | n.d. |
| | | | 5 i.v. | 12.7 | 5 min | 7.6 (7.1–8.1) | 105 | 72±2 |
| | | | 25 oral | 46.5 | 3 h | 9.7 (8.9–10.6) | 804 | 53 |
| D | 1 | 20 | 12.5 oral | 13.1 | 12 h | 19.4 (17.3–22.0) | 450 | n.d. |
| | | | 5 i.v. | 12.4 | 5 min | 10.2 (9.2–11.4) | 138 | 74±1 |
| E | 2 | 9 | 50 oral | 59.8 | 5 h | 5.7 (5.3–6.1) | 760 | 55±3 |
| F | 2 | 10 | 50 oral | 54.8 | 3 h | 6.8 (6.4–7.3) | 707 | 52±3 |

[a]Where binding was determined over the full-time course, values given are the mean±s.e. ($n=8$). Where no errors are shown, results are the mean of the 4 and 5 h values only.
[b]Samples were collected up to 9 h only.
[c]1 – Collie crossbred; 2 – Beagle.

obtained for a precise calculation of the distribution phase half life ($t\frac{1}{2}\alpha$) but this was estimated by back-stripping to be about 10–25 min. The elimination phase half-life ($t\frac{1}{2}\beta$) was comparable to that for the oral route (Table I). Plasma clearance values for dogs C and D were 0.0476 and 0.0362 $l\,h^{-1}\,kg^{-1}$ respectively. After normalising for dose, comparison of $AUC_{0-\infty}$ data for oral administration with those for i.v. data in the same dogs gave an overall bioavailability of about 130%: i.e., the availability was greater with the oral route.

The volume of distribution ($Vd_{area}$) was calculated from the total plasma i.v. data to be 0.52 and 0.53 $l\,kg^{-1}$ for dogs C and D respectively; the corresponding values calculated from unbound drug data were 2.05 and 1.87 $l\,kg^{-1}$.

*Sheep* Two sheep received an i.v. dose of 4 $mg\,kg^{-1}$. Protein binding (±s.e., $n=6$) was 41 ±2% and 42±2% respectively. For whole plasma benznidazole, respective peak concentrations of 3.6 and 2.5 $\mu g\,ml^{-1}$ were obtained, and the elimination $t\frac{1}{2}$ values (with 95% confidence limits) were 5.2 (4.1–6.9) h and 3.8 (2.6–7.2) h.

### *Urinary excretion*

Benznidazole was given i.p. to groups of five BALB/c mice at a dose of 650 $mg\,kg^{-1}$ and the 24 h urinary recoveries determined. Values obtained in two experiments were 4.8 and 5.2% respectively.

### *Tumour and normal tissue penetration*

*Mice* The data on the penetration of benznidazole into the EMT6 and KHT mouse tumours are summarised in Table II. Benznidazole was administered either as a single i.p. dose with sampling at 45, 75, 105 and 135 min for EMT6 (Brown & Workman, 1980), or in multiple i.p. doses with sampling between 1 and 16 h for KHT (Twentyman & Workman, 1983). The mean plasma concentration did not vary by more than a factor of two over the sampling period (Table II). For simplicity the data are presented as tumour/whole plasma ratios averaged over the entire sampling period for each experiment. However, it should be noted that whereas with KHT the ratios were constant, with the shorter sampling period used for EMT6 there was a tendency for the ratios to increase with time. For example, in experiment b (Table II) the tumour/whole plasma ratios (s.e., $n=5$) at 45, 75, 105 and 135 min were 58±8%, 70±5%, 97±4% and 88±9%, respectively. Thus although the overall mean ratios in Table II are lower for EMT6 than for KHT, the actual steady-state values were very similar indeed.

In the second experiment with the KHT tumour,

**Table II** Penetration of benznidazole into mouse tumours.

| Tumour (site) | Tumour/whole plasma ratio (% ± s.e.) Exp 1 | Exp 2 |
|---|---|---|
| EMT6 (i.d. flank)[1] | 59±9[a] (n=16) | 79±5[b] (n=20) |
| EMT6 (i.m. leg)[1] | 75±5[c] (n=20) | 74±6[d] (n=20) |
| KHT (i.m. leg)[2] | 99±3[e] (n=25) | 90±3[f] (n=13) |

[a]Single dose of 650 mg kg$^{-1}$ i.p.; sampling times 45, 75, 105 and 135 min (Brown & Workman, 1980); range of mean plasma concentrations 49–99 μg ml$^{-1}$.
[b]Dosing as [a]; range of mean plasma concentrations 77–121 μg ml$^{-1}$.
[c]Dosing as [a]; range of mean plasma concentrations 100–130 μg ml$^{-1}$.
[d]Dosing as [a]; range of mean plasma concentrations 74–133 μg ml$^{-1}$.
[e]Priming dose of 60 mg kg$^{-1}$ i.p. followed by 15 hourly doses of 15 mg kg$^{-1}$ i.p. (Twentyman & Workman, 1983); sampling times 1, 4, 8, 11 and 16 h; range of mean plasma concentrations 25–47 μg ml$^{-1}$.
[f]Dosing as [e] but omitting the dose at 1 h; sampling times 2, 4, 7, 8, 12 and 16 h; range of mean plasma concentrations 12–29 μg ml$^{-1}$.
[1]BALB/c mice.
[2]C3H/He mice.

ratios were also determined at 1 h following single i.p. doses of 78 and 260 mg kg$^{-1}$: these were respectively 88±5% and 88±2% (s.e., $n=5$), which compare closely with the ratio of 90±3% ($n=13$) for the multiple dose schedule.

As far as absolute tumour concentrations are concerned, a single i.p. dose of 650 mg kg$^{-1}$ gave peak levels of 55±11 μg g$^{-1}$ (s.e., $n=4$) and 104 ±10 μg g$^{-1}$ ($n=5$) for the two experiments with EMT6 i.d. flank tumours, and 130±7 μg g$^{-1}$ ($n=5$) and 84±14 μg g$^{-1}$ ($n=5$) for the two with EMT6 i.m. leg tumours. (The lower level in the first flank experiment was due to administration as a suspension in arachis oil, which gives a correspondingly lower plasma concentration than 50% polyethylene glycol). With the multiple dose schedule steady-state concentrations in the KHT tumour were 26 and 32 μg g$^{-1}$ respectively.

In three of the experiments where single i.p. doses of 650 mg kg$^{-1}$ were given to BALB/c mice bearing EMT6 tumours, whole brain concentrations of benznidazole were also determined at the same time (Brown & Workman, 1980). Overall mean brain/whole plasma ratios are presented in Table III, but, as for the EMT6 tumour, these tended to increase over the sampling period: in experiment b, for example, the ratios (±s.e., $n=5$) at 45, 75, 105 and 135 min were 56±3%, 69±3%, 71±5% and 75 ±5%, respectively. Brain/whole plasma ratios and absolute brain concentrations were similar to those for the EMT6 tumour in the same mice. Tumour/brain ratios averaged between 99 and 120% (Table III).

**Table III** Penetration of benznidazole into whole brain of BALB/c mice bearing EMT6 tumours.

| | Brain/whole plasma ratio (% ± s.e.) | Tumour/brain ratio (% ± s.e.) |
|---|---|---|
| Exp 1[a] | 61±5 (n=16) | 120±33 (n=16) |
| Exp 2[b] | 68±3 (n=20) | 116±5 (n=20) |
| Exp 3[c] | 76±4 (n=20) | 99±7 (n=20) |

The superscripts [a,b] and [c] refer to footnotes shown in **Table II**, and allow comparison with the tumour/plasma ratios from the same experiment.

Three experiments were carried out to compare liver and whole plasma concentrations 0.5, 3 and 6 h after an i.p. dose of 78 mg kg$^{-1}$ (Table IV). Liver/whole plasma ratios were fairly constant over this period, averaging 42±4% (s.e., $n=5$) in BALB/c mice and 71±8% ($n=29$) in C3H/He mice.

*Dogs* Benznidazole was administered orally at a dose of 12.5 mg kg$^{-1}$ or, in one case, 25 mg kg$^{-1}$ to seven dogs bearing spontaneous neoplasms. Whole plasma concentrations and protein binding were similar to the results described earlier for normal dogs. The tissue samples were taken between 1.67 and 4.5 h, the period when peak concentrations usually occur (Table I), and the tissue benznidazole concentrations and tissue/whole plasma ratios are given in Table V. Most of the samples were from primary or secondary tumour deposits, but in some cases apparently normal tissues were also obtained.

In the case of the neoplastic tissues, penetration of benznidazole was generally good, with tissue/whole plasma ratios ranging from 14–70%. The overall mean±s.e. for the 24 samples (excluding cyst fluid) was 50±3%, and most of the values were reasonably close to this figure. Some of the lowest ratios were obtained for the mixed mammary tumour (dog 4), which contained particularly tough tissue; especially low ratios were noted in the cystic area and cyst fluid. The spleen metastasis of the metastatic mammary adenocarcinoma also had a low ratio. Inner and outer areas of tumour were analysed in dogs 2 and 4, but there was no consistent pattern. In terms of

**Table IV** Penetration of benznidazole (78 $mg\,kg^{-1}$ i.p.) into mouse liver.

| | *BALB/c* | | | *C3H/He* | | |
|---|---|---|---|---|---|---|
| *Time* (*h*) | *Whole plasma* ($\mu g\,ml^{-1}$) | *Liver* ($\mu g\,g^{-1}$) | *Liver/whole plasma* (%) | *Whole plasma* ($\mu g\,ml^{-1}$) | *Liver* ($\mu g\,g^{-1}$) | *Liver/whole plasma* (%) |
| 0.5 | 45.3 ±3.3 (n=5) | 20.8 ±3.3 (n=5) | 46.6 ±7.2 (n=5) | 33.9 ±2.2 (n=10) | 25.2 ±2.2 (n=10) | 75.8 ±7.3 (n=10) |
| 3.0 | 29.8 ±1.9 (n=5) | 10.3 ±1.8 (n=5) | 34.8 ±6.4 (n=5) | 34.1 ±1.6 (n=10) | 17.3 ±3.7 (n=10) | 53.3 ±12.2 (n=10) |
| 6.0 | 12.2 ±3.3 (n=5) | 8.2 ±1.6 (n=5) | 43.9 ±5.9 (n=5) | 21.1 ±1.2 (n=10) | 17.8 ±3.3 (n=9) | 84.3 ±17.6 (n=9) |
| Overall | | | 41.7 ±3.7 (n=15) | | | 70.6 ±7.5 (n=29) |

Results are expressed as the mean±s.e. of *n* determinations. BALB/c data are from one experiment; C3H data pooled from two experiments.

absolute neoplastic tissue concentrations, the overall mean±s.e. for the 24 samples taken between 1.67 and 4.5 h was 5.6±0.5 $\mu g\,g^{-1}$ (values from dog 3 were halved to normalise to a dose of 12.5 $mg\,kg^{-1}$).

Although a low value was obtained for liver in dog 6, the results for normal tissues were generally similar to those for tumours. This included the brain and peripheral nerve samples from dog 6. A more extensive study of benznidazole penetration into brain and peripheral nerve was carried out in two non-tumour bearing dogs, and the results are shown in Table VI. Plasma concentrations were rather low for the oral dose of 25 $mg\,kg^{-1}$, particularly in one dog, and this was probably a result of the general anaesthetic. Brain/whole plasma ratios were fairly constant over the 1–4 h period, with an overall mean of 42±3% (s.e., $n=8$). Peripheral nerve/whole plasma ratios tended to increase over the sampling period and the mean of 74±7% (s.e., $n=7$) was higher than that for brain.

### Discussion

Although, compared with MISO, it exhibits similar or slightly greater radiosensitization of hypoxic cells (Adams *et al.*, 1979; Anderson & Patel, 1979), the clinical potential of benznidazole in cancer treatment is as a chemosensitizer, particularly in combination with CCNU. We have shown that to obtain a given enhancement of tumour response to CCNU the benznidazole dose required is much lower than that for misonidazole (Workman & Twentyman, 1982); moreover, the tumour enhancement is greater than that seen in normal tissues and the therapeutic gain with low dose benzidazole is similar to that with high dose MISO (Twentyman & Workman, 1983). Very similar results were reported by Siemann *et al.* (1983). It should be noted, however, that in multiple dose experiments designed to simulate human pharmacokinetics, Hirst *et al.* (1983) found that the therapeutic gain with benznidazole was in certain instances inferior to that for MISO.

When combined with melphalan benznidazole gave greater enhancement of tumour response than MISO but the evidence for a therapeutic gain was equivocal (Sheldon & Batten, 1982; Twentyman & Workman, 1983). With cyclophosphamide no enhancement was seen by Twentyman & Workman (1982), or McNally (personal communication), but enhancement was observed by Chaplin *et al.* (1984 not published).

Reviewing the chemosensitization data overall, we felt that the combination of benznidazole plus CCNU showed the most promise for clinical use (Twentyman & Workman, 1983). Particularly important was the greater potency of benznidazole compared to MISO, which suggested that nitroimidazole neurotoxicity might be avoided. Moreover, prolonged daily administration of benznidazole had been used to treat South American patients with the trypanosomal infection Chagas' disease or with mucotaneous Leishmaniasis (Barclay *et al.*, 1978; Cerisola *et al.*, 1978; Coura *et al.*, 1978; Fava *et al.*, 1978). The main objectives of the present communication are: (i) to describe the detailed pharmacokinetics of benznidazole; (ii) to

**Table V** Penetration of benznidazole into spontaneous tumours and certain normal tissues in dogs.

| *Dog* | *Weight (kg)* | *Dose (mg kg$^{-1}$)* | *Tumour* | *Sample* | *Time (h)* | *Concentration (μg g$^{-1}$) (tissue/plasma %)* |
|---|---|---|---|---|---|---|
| 1 | 36 | 12.5 | Fibroleiomyoma | Primary | 2.3 | 4.2 (40%); 4.3 (41%); 5.5 (52%) |
| 2 | 28 | 12.5 | Lymphoblastic lymphosarcoma | Lymph node | | |
| | | | | (a) Outer area | | 2.1 (43%) |
| | | | | (b) Intermediate area | 3.0 | 3.0 (60%) |
| | | | | (c) Centre | | 2.3 (46%) |
| 3 | 7 | 25 | Metastatic mammary adenocarcinoma | (a) Primary | | 12.8 (65%) |
| | | | | (b) Lung metastasis | 1.67 | 16.7 (85%) |
| | | | | (c) Spleen metastasis | | 5.6 (29%) |
| 4 | 12 | 12.5 | Mixed mammary tumour | (a) First tumour: outer area | | 4.2 (46%) |
| | | | | (b) First tumour: centre | | 2.8 (31%) |
| | | | | (c) First tumour: cystic area | 4.5 | 1.2 (14%) |
| | | | | (d) First tumour: cyst fluid | | 2.1 (23%) |
| | | | | (e) Second tumour | | 5.4 (59%) |
| | | | | (f) Normal skin | | 5.9 (65%) |
| 5 | 14 | 12.5 | Lymphoblastic lymphosarcoma | Lymph node | 2.25 | 4.8 (55%); 4.7 (53%) |
| 6 | 29 | 12.5 | Osteosarcoma (9th rib) | (a) Tumour | | 9.4 (46%); 8.8 (43%) |
| | | | | (b) Normal brain | | 8.9 (44%); 9.5 (46%) |
| | | | | (c) Normal peripheral nerve | 4.0 | 7.6 (37%) |
| | | | | (d) Normal liver | | 2.8 (14%) |
| 7 | 47 | 12.5 | Oral anaplastic sarcoma | (a) Primary | | 9.9 (70%) |
| | | | | (b) Rib metastasis | | 8.9 (63%) |
| | | | | (c) Cervical vertebra metastasis | | 9.1 (65%) |
| | | | | (d) Normal kidney | 4.5 | 6.2 (44%) |
| | | | | (e) Normal pancreas | | 7.6 (54%) |
| | | | | (f) Normal lung | | 7.9 (56%) |
| | | | | (g) Normal liver | | 4.1 (51%) |

**Table VI** Penetration of benznidazole into brain and peripheral nerve in two crossbred dogs given an oral dose of $25\,mg\,kg^{-1}$.

| | *Benznidazole concentration* ($\mu g\,ml^{-1}$ *or* $\mu g\,g^{-1}$) *(tissue/plasma ratio %)* | | | | | |
|---|---|---|---|---|---|---|
| | *Dog G* | | | *Dog H* | | |
| *Time (h)* | Plasma | Brain | Peripheral nerve | Plasma | Brain | Peripheral nerve |
| 1 | 3.1 | 1.1 (35%) | 1.3 (41%) | 23.1 | 11.6 (50%) | 14.0 (60%) |
| 2 | 3.8 | 1.5 (39%) | 3.4 (90%) | 22.2 | 10.0 (45%) | 15.5 (70%) |
| 3 | 4.3 | 2.1 (49%) | n.d. | 18.7 | 8.5 (45%) | 14.7 (78%) |
| 4 | 4.1 | 1.1 (26%) | 4.0 (96%) | 18.5 | 7.9 (43%) | 15.6 (84%) |

n.d. = not determined.

relate the pharmacokinetic behaviour to chemosensitization; and (iii) to illustrate how this data base has been used for the estimation of the benznidazole dose which might produce chemosensitization in man.

Benznidazole showed considerable binding to plasma proteins in all species studied: 39% in the mouse, 59% in the dog, and 42% in the sheep. These figures are comparable with those of 58% (Workman & Brown, 1981) and 44% (Raaflub & Ziegler, 1979) for human plasma and of 46% for bovine serum albumin (Clarke & Wardman, cited in Watts *et al.,* 1980). Binding is probably to hydrophobic sites on proteins, but hydrogen bonding through the amide group may also occur (Watts *et al.,* 1980).

After i.v. administration to mice and dogs benznidazole was cleared biphasically. However the distribution phase was extremely short, and negligible error would be introduced by the use of the one-compartment model to calculate drug clearance. With oral administration in the dog peak plasma concentrations were attained rapidly (usually 1–5 h) and bioavailability was complete. Similar bioavailability was also seen with i.p. administration in mice, and peak concentrations were usually achieved by 30 min.

Pharmacokinetics were linear up to doses of $78\,mg\,kg^{-1}$ in mice, but became non-linear above this. For example, at $650\,mg\,kg^{-1}$ peak plasma concentrations ($100\,\mu g\,ml^{-1}$) were far less than predicted from lower doses, and were maintained to at least 6 h before declining slowly. Because of its low solubility in aqueous solution benznidazole was usually given as a suspension in 50% polyethylene glycol, and this undoubtedly limited the absorption rate and contributed to the slow clearance at high doses. There may also be saturation of hepatic metabolism, as seen with other lipophilic nitroimidazoles (Workman & Brown, 1981). Since only 5% of administered benznidazole was recovered unchanged in the urine, the predominant elimination mechanism is likely to be metabolism although other mechanisms (e.g. biliary and faecal excretion) cannot be excluded. Schwartz *et al.* (unpublished) obtained evidence for the 2-hydroxy "hydrolysis" product and the 2-amino reduction product (see Schwartz & Hofheinz, 1982), and we have also identified the amine metabolite (Walton & Workman, in preparation). By analogy with other nitroimidazoles it is likely that ring cleavage also occurs (Schwartz & Hofheinz, 1982).

Tumour penetration by benznidazole was generally good. For the transplantable tumours in mice (KHT and EMT6) the average tumour/whole plasma ratios ranged from 59–99%, and steady-state ratios were about 90%. With the spontaneous neoplasms in dogs tissue/whole plasma ratios ranged from 14–70% and the overall mean was 50%. Nervous tissue penetration was generally similar to that in tumours. Brain/whole plasma ratios averaged 61–76% in the mouse and 42% in the dog, while the mean peripheral nerve/whole plasma ratio in the dog was 74%. Liver/whole plasma ratios averaged 42 and 71% in the two mouse strains studied, while the two values obtained in the dog were 14 and 51%.

It may be useful to compare briefly the pharmacokinetics of benznidazole with those of the more familiar MISO (e.g. see Workman, 1980*b* and 1983). Although similar in electron affinity (redox potential) benznidazole is considerably more lipophilic (Adams *et al.,* 1979), and lipophilicity has a major effect on nitroimidazole pharmacokinetics (see Workman, 1982*b*). Both exhibit non-linear kinetics at very high doses in mice. At lower doses both are absorbed quite rapidly after i.p. and oral administration in mice and dogs, respectively. Peak concentrations per unit dose were similar in mice, and similar or higher for benznidazole in dogs. The elimination $t\frac{1}{2}$ was longer for benznidazole in all three species: 90 min compared with 20–40 min in mice; 4–5 h compared with 40–60 min in sheep (Miller, personal communication); and 9–11 h compared with 4–5 h in large crossbred dogs. The difference does not appear so large in humans in whom Raaflub & Ziegler (1979) and Raaflub (1980), using polarographic analysis after low doses, found mean $t\frac{1}{2}$ values of 12–14 h, only slightly longer than the usual 10–12 h average for MISO. Both compounds are eliminated predominantly by metabolism. The volume of distribution for benznidazole in the dog was $0.53 \, l\,kg^{-1}$, which compares well with $0.62 \, l\,kg^{-1}$ for MISO.

Tissue/whole plasma ratios tended to be rather lower for benznidazole, possibly as a consequence of its appreciable protein binding (39–59%) which does not occur with MISO. The difference was most obvious in the brain where the ratios for benznidazole were 42% in dogs and 68% in mice compared to respective values of 70% and 90% for MISO. In EMT6 flank tumours the overall tissue/whole plasma ratio was 69% for benznidazole compared to 87% for MISO, but the steady-state ratio was about 90% for both; in spontaneous dog neoplasms the values were 50% for benznidazole and 61% for MISO. The difference was least in dog peripheral nerve (74% for benznidazole and 82% for MISO).

We have shown recently that for the combination of CCNU with misonidazole a major mechanism appears to involve the inhibition of CCNU metabolism by the sensitizer, probably in the liver, resulting in elevated CCNU concentrations in tumour but not normal tissues (Lee & Workman, 1983, 1984*a*). Benznidazole slows CCNU clearance at much lower doses than misonidazole (Lee & Workman, 1984*b*), is a considerably more potent inhibitor of drug metabolising enzymes *in vivo* (Workman *et al.*, 1983) and exhibits more powerful inhibition of CCNU hydroxylation by liver microsome preparations *in vitro* (Lee & Workman, unpublished): this explains the comparative potency of benznidazole as a chemosensitizer with CCNU. There may also be the additional mechanisms of chemosensitization which require the presence of the nitroimidazole in the tumour (see Brown, 1982; Siemann, 1982). Whatever the mechanism, for the clinical application of the benznidazole-CCNU combination we should aim for whole plasma, liver and tumour concentrations of nitroimidazole similar to those associated with chemosensitization in mice.

Clear enhancement of KHT tumour response to CCNU can be obtained with i.p. benznidazole doses at least as low as $13\,mg\,kg^{-1}$ (Workman & Twentyman, 1982) which would produce peak whole plasma, liver and tumour concentrations of about $10\,\mu g\,ml^{-1}$, $7\,\mu g\,g^{-1}$ and $9\,\mu g\,g^{-1}$, respectively. Between 26 and $650\,mg\,kg^{-1}$ the dose-response curve becomes flat, with comparatively little gain in chemosensitization at increasing doses; this is almost certainly because the dose-peak whole plasma concentration curve has the same shape (Figure 4), with the plasma concentration increasing by a factor of only four (from $20\,\mu g\,ml^{-1}$ to $90\,\mu g\,ml^{-1}$) over the 25-fold dose range. The most detailed chemosensitization work in mice has been done with an i.p. benznidazole dose of $78\,mg\,kg^{-1}$, which gives whole plasma, liver and tumour concentrations of $30\,\mu g\,ml^{-1}$, $21\,\mu g\,g^{-1}$ and $26\,\mu g\,g^{-1}$, respectively. At this dose tumour response is enhanced by a factor of 1.5–2 compared to 1.2–1.4 in normal tissues, resulting in a net therapeutic gain (Twentyman & Workman, 1983; Siemann *et al.*, 1983). On the other hand, the results of Hirst *et al.* (1983) would indicate that this therapeutic gain can be reduced when benznidazole whole plasma concentrations are increased to around $100\,\mu g\,ml^{-1}$. Taken overall these data suggest that for chemosensitization by benznidazole in man we should probably aim for peak whole plasma concentrations in the range $10–30\,\mu g\,ml^{-1}$.

Because of its low aqueous solubility (and also for convenience) benznidazole will be administered orally in man. In large crossbred dogs we achieved average peak whole plasma concentrations of 20 and $47\,\mu g\,ml^{-1}$ with oral doses of 12.5 and $25\,mg\,kg^{-1}$ (Table I). For each $mg\,kg^{-1}$ administered the mean peak plasma concentration was $1.77 \pm 0.17\,\mu g\,ml^{-1}$ (s.e., $n=6$), which compares favourably with the value of $1.48 \pm 0.06$ (s.e., $n=7$) obtained with oral administration at comparatively low doses (mean $1.73\,mg\,kg^{-1}$) in man (Raaflub & Ziegler, 1979). Thus we predict that the target concentrations of $10–30\,\mu g\,ml^{-1}$ would be achieved with oral doses of $6–20\,mg\,kg^{-1}$ in man. In the South American studies with benznidazole as an antimicrobial agent, doses of $3–10\,mg\,kg^{-1}$ were given for 30–60 days (Barclay *et al.*, 1978; Cerisola *et al.*, 1978; Coura *et al.*, 1978; Fava *et al.*, 1978). With an average daily dose of $3.5\,mg\,kg^{-1}$ the mean steady state minimum and maximum plasma concentrations were $8.3 \pm 0.6\,\mu g\,ml^{-1}$ and $12.4 \pm 0.7\,\mu g\,ml^{-1}$ (s.e., $n=6$) respectively. Side effects were observed, including peripheral neuropathy, but the schedule of $5\,mg\,kg^{-1}\,day^{-1}$ for 30 days was considered well tolerated. For chemosensitization purposes benznidazole would be administered only intermittently with each CCNU cycle, and peripheral neuropathy should be avoided.

As far as the relative timing of CCNU and benznidazole is concerned, our mouse studies have shown that no advantage is gained by prolonged exposure to the sensitizer (Twentyman & Workman, 1983). We obtained peak concentrations

3–5 h after oral dosing in dogs, which compares favourably with the peak time of 3–4 h after low doses in man (Raaflub & Ziegler, 1979).

A phase I clinical trial of benznidazole plus CCNU, with associated pharmacokinetic studies, is now underway in this Unit. Designed with the above considerations in mind, this involves escalating oral doses of benznidazole, commencing at $8\,mg\,kg^{-1}$, which are given 4 h before $120\,mg\,m^{-2}$ CCNU orally once every 6 weeks.

We thank Dr Carey Smithen of Roche Products Ltd. for supplies of benznidazole and Ro 07-0913, and Jane Donaldson and Nancy Smith for excellent technical assistance.

### References

ADAMS, G.E., CLARKE, E.D., FLOCKHART, I.R. & 8 others. (1979). Structure-activity relationships in the development of hypoxic cell radiosensitizers. I. Sensitization efficiency. *Int. J. Radiat. Biol.*, **35**, 133.

ANDERSON, R.F. & PATEL, K.B. (1979). Effect of lipophility of nitroimidazoles on radiosensitization of hypoxic bacterial cells *in vitro*. *Br. J. Cancer*, **39**, 705.

BARCLAY, C.A., CERISOLA, J.A., LUGONES, H. & LEDESMA, O. (1978). Status of the clinical and seroparasitological evaluation of benznidazole in the treatment of acute Chagas' disease. In: *Current Chemotherapy*. (Eds. Siegenthaler & Lathy), Washington: America Society for Microbiology, vol. 1, p. 158.

BROWN, J.M. (1982). The mechanism of cytotoxicity and chemosensitization by misonidazole and other nitroimidazoles. *Int. J. Radiat. Oncol. Biol. Phys.*, **8**, 675.

BROWN, J.M. & WORKMAN, P. (1980). Partition coefficient as a guide to the development of radiosensitizers which are less toxic than misonidazole. *Radiat. Res.*, **82**, 171.

CERISOLA, J.A., BARCLAY, C.A., SILVA, J.L. & MOUZO, G. (1978). Anti-*Trypanosoma cruzi* activity of benznidazole in chronic Chagas' infection. In: *Current Chemotherapy*. (Eds. Siegenthaler & Lathy), Washington: American Society for Microbiology, vol. 1, p. 159.

COURA, J.R., BRINDEIRO, P.J. & FERREIRA, I. (1978). Benznidazole in the treatment of Chagas' disease. In: *Current Chemotherapy*. (Eds. Siegenthaler & Lathy), Washington: American Society for Microbiology, vol. 1, p. 161.

DISCHE, S., SAUNDERS, M.I., LEE, M.E., ADAMS, G.E. & FLOCKHART, I.R. (1977). Clinical testing of the radiosensitizers Ro 07-0582: Experience with multiple doses. *Br. J. Cancer*, **35**, 567.

FAVA, S.DiC., ZAMITH, V.A., CUCE, L.C. & SAMPAIO, S.A. (1978). Treatment of American mucotaneous Leishmaniasis with benznidazole. In: *Current Chemotherapy*. (Eds. Siegenthaler & Lathy), Washington: American Society for Microbiology, vol. 1, p. 163.

HIRST, D.G., BROWN, J.M. & HAZLEHURST, J.L. (1983). Effect of partition coefficient on the ability of nitroimidazoles to enhance the cytotoxicity of 1-(2-chloroethyl)3-cyclohexyl-nitrosourea. *Cancer Res.*, **43**, 1961.

LEE, F.Y.F. & WORKMAN, P. (1983). Modification of CCNU pharmacokinetics by misonidazole – a major mechanism of chemosensitization in mice. *Br. J. Cancer*, **47**, 659.

LEE, F.Y.F. & WORKMAN, P. (1984*a*). Misonidazole and CCNU: Further evidence for a pharmacokinetic mechanism of chemosensitization and therapeutic gain. *Br. J. Cancer*, **49**, 579.

LEE, F.Y.F. & WORKMAN, P. (1984*b*). Nitroimidazoles as modifiers of nitrosourea pharmacokinetics. *Int. J. Radiat. Oncol. Biol. Phys.* (In press).

McNALLY, N.J. (1982). Enhancement of chemotherapy agents. *Int. J. Radiat. Oncol. Biol. Phys.*, **8**, 593.

RAAFLUB, J. (1980). Multi-dose kinetics of the trypanosomicide benznidazole in man. *Arzneimittel-forsch.*, **30**, 2192.

RAAFLUB, J. & ZIEGLER, W.H. (1979). Single-dose pharmacokinetics of the trypanosomicide benznidazole in man. *Arzneimittel-forsch.*, **29**, 1611.

SCHWARTZ, D.E. & HOFHEINZ, W. (1982). Metabolism of nitroimidazoles. In: *Nitroimidazoles. Chemistry, Pharmacology and Clinical Application*. (Eds. Breccia *et al.*), Nato Advanced Study Institute Series, Series A: *Life Sci.*, **42**, 189.

SHELDON, P.W. & BATTEN, E.L. (1982). Potentiation *in vivo* of melphalan activity by nitroimidazole compounds. *Int. J. Radiol. Oncol. Biol. Phys.*, **8**, 635.

SIEMANN, D.W. (1982). Potentiation of chemotherapy by hypoxic cell radiation sensitizers. *Int. J. Radiat. Oncol. Biol. Phys.*, **8**, 1029.

SIEMANN, D.W., MORRISEY, S. & WOLF, K. (1983). *In vivo* potentiation of 1-(2-chloroethyl)-3-cyclohexyl-1-nitrosourea by the radiation sensitizer benznidazole. *Cancer Res.*, **43**, 1010.

TWENTYMAN, P.R., KALLMAN, R.F. & BROWN, J.M. (1979). The effect of time between X-irradiation and chemotherapy on the growth of three solid mouse tumours – I. Adriamycin. *Int. J. Radiat. Oncol. Biol. Phys.*, **5**, 1255.

TWENTYMAN, P.R. & BLEEHEN, N.M. (1975). Studies of "potentially lethal damage" in EMT6 mouse tumour cells treated with bleomycin either *in vitro* or *in vivo*. *Br. J. Cancer*, **32**, 491.

TWENTYMAN, P.R. & WORKMAN, P. (1983). Chemosensitization by lipophilic nitroimidazoles. *Br. J. Cancer*, **48**, 17.

WATTS, M.E., ANDERSON, R.F., JACOBS, R.S. & 7 others (1980). Evaluation of novel hypoxic cell radiosensitizers *in vivo*. In: *Radiation Sensitizers*. (Ed. Brady), New York: Masson, p. 175.

WHITE, R.A.S. & WORKMAN, P. (1980). Pharmacokinetics and tumour-penetration properties of the hypoxic cell radiosensitizer desmythylmisonidazole (Ro 05-9963) in dogs. *Br. J. Cancer*, **41**, 268.

WHITE, R.A.S., WORKMAN, P. & BROWN, J.M. (1980). The pharmacokinetics and tumour and neural tissue penetrating properties of SR-2508 and SR-2555-hydrophilic radiosensitizers potentially less toxic than misonidazole. *Radiat. Res.*, **841**, 542.

WHITE, R.A.S., WORKMAN, P. & OWEN, L.N. (1982). The pharmacokinetics in mice and dogs of nitroimidazole radiosensitizers and chemosensitizers more lipophilic than misonidazole. *Int. J. Radiat. Oncol. Biol. Phys.*, **8**, 473.

WHITE, R.A.S., WORKMAN, P., OWEN, L.N. & BLEEHEN, N.M. (1979). The penetration of misonidazole into spontaneous canine tumours. *Br. J. Cancer*, **40**, 284.

WORKMAN, P. (1980*a*). Dose-dependence and related studies on the pharmacokinetics of misonidazole and demethylmisonidazole in mice. *Cancer Chemother. Pharmacol.*, **5**, 27.

WORKMAN, P. (1980*b*). Pharmacokinetics of hypoxic cell radiosensitizers: A review. In: *Radiation Sensitizers*. (Ed. Brady), New York: Masson, p. 192.

WORKMAN, P. (1982). Lipophility and the pharmacokinetics of nitroimidazoles. In: *Advanced Topics on Radiosensitizers of Hypoxic Cells*. (Eds. Breccia *et al.*), Nato Advanced Study Institute Series, Series A: *Life Sci.*, **43**, 143.

WORKMAN, P. (1983). Pharmacokinetics of radiosensitizing agents. In: *Pharmacokinetics of Anticancer Agents in Humans*. (Eds. Ames *et al.*), Amsterdam: Elsevier, p. 291.

WORKMAN, P. & BROWN, J.M. (1981). Structure-pharmacokinetic relationships for misonidazole analogues in mice. *Cancer Chemother. Pharmacol.*, **6**, 39.

WORKMAN, P. & TWENTYMAN, P.R. (1982). Structure/activity relationships for the enhancement by electron-affinic drugs of the anti-tumour effect of CCNU. *Br. J. Cancer*, **46**, 249.

WORKMAN, P., LITTLE, C.J., MARTEN, T.R. & 4 others (1978). Estimation of the hypoxic cell sensitizer misonidazole and its O-demethylated metabolite in biological materials by reversed-phase high-performance liquid chromatography. *J. Chromatogr.*, **145**, 507.

WORKMAN, P., TWENTYMAN, P.R., LEE, F.Y.F. & WALTON, M.I. (1983). Drug metabolism and chemosensitization. Nitroimidazoles as inhibitors of drug metabolism. *Biochem. Pharmacol.*, **32**, 857.